# Investigation of the Acetylation Mechanism by GCN5 Histone Acetyltransferase

**DOI:** 10.1371/journal.pone.0036660

**Published:** 2012-05-04

**Authors:** Junfeng Jiang, Junyan Lu, Dan Lu, Zhongjie Liang, Lianchun Li, Sisheng Ouyang, Xiangqian Kong, Hualiang Jiang, Bairong Shen, Cheng Luo

**Affiliations:** 1 Center for Systems Biology, Soochow University, Jiangsu, China; 2 Drug Discovery and Design Center, State Key Laboratory of Drug Research, Shanghai Institute of Materia Medica, Chinese Academy of Sciences, Shanghai, China; Institute of Genetics and Molecular and Cellular Biology, France

## Abstract

The histone acetylation of post-translational modification can be highly dynamic and play a crucial role in regulating cellular proliferation, survival, differentiation and motility. Of the enzymes that mediate post-translation modifications, the GCN5 of the histone acetyltransferase (HAT) proteins family that add acetyl groups to target lysine residues within histones, has been most extensively studied. According to the mechanism studies of GCN5 related proteins, two key processes, deprotonation and acetylation, must be involved. However, as a fundamental issue, the structure of hGCN5/AcCoA/pH3 remains elusive. Although biological experiments have proved that GCN5 mediates the acetylation process through the sequential mechanism pathway, a dynamic view of the catalytic process and the molecular basis for hGCN5/AcCoA/pH3 are still not available and none of theoretical studies has been reported to other related enzymes in HAT family. To explore the molecular basis for the catalytic mechanism, computational approaches including molecular modeling, molecular dynamic (MD) simulation and quantum mechanics/molecular mechanics (QM/MM) simulation were carried out. The initial hGCN5/AcCoA/pH3 complex structure was modeled and a reasonable snapshot was extracted from the trajectory of a 20 ns MD simulation, with considering post-MD analysis and reported experimental results. Those residues playing crucial roles in binding affinity and acetylation reaction were comprehensively investigated. It demonstrated Glu80 acted as the general base for deprotonation of Lys171 from H3. Furthermore, the two-dimensional QM/MM potential energy surface was employed to study the sequential pathway acetylation mechanism. Energy barriers of addition-elimination reaction in acetylation obtained from QM/MM calculation indicated the point of the intermediate ternary complex. Our study may provide insights into the detailed mechanism for acetylation reaction of GCN5, and has important implications for the discovery of regulators against GCN5 enzymes and related HAT family enzymes.

## Introduction

The post-translational modification of histones has been reported playing crucial roles in chromatin regulation. It insures the fidelity of opened chromatin structure, increased gene expression and other DNA transactions [1], [2]. Involved in DNA recognition by transcription factors and access of genetic information, histone modification is one of the most important processes to obtain an open chromatin structure and/or to recruit specific proteins and thus influence gene expression, DNA replication and repair, and chromosome condensation and segregation. These “epigenetic” changes can be highly dynamic and play a crucial role in regulating cell proliferation, survival, differentiation and motility. Among different epigenetic modifications, the increased global histone acetylation degree always correlates with transcriptional regulation in euchromatin and heterochromatin [3], [4], [5], [6], in which gene transcription levels are changed during early stem cells differentiation in a tissue specific manner. Since altered epigenetic modifications play key roles in kinds of diseases, an intense attention should be paid for the players that adding or removing of these epigenetic markers because of their roles as potential “druggable” therapeutic targets.

Each core histone protein possesses a globular domain and a long N-terminal tails rich in lysine residues. Therefore the histone proteins are positively charged under physiological condition and can be covalently modified, which has been regarded as “identification signal” for transcriptional regulation [7]. After proton moves away, acetylation of lysine tails on histones causes weaker binding of nucleosome to DNA. Moreover, the added acetyl group neutralizes the positive charges of histone proteins, which could always generate a more relaxed open transcription-permissive structure [8], [9]. This mechanism induces the exposure of chromatin structure [10], enabling the binding of transcription factors and significantly increasing gene expression [11]. Similar to DNA methylation, histone acetylation has been reported playing a significant role in epigenetic memory and stem cell differentiation [12], [13]. The increasing degree of histone acetylation during somatic cell reprogramming may indicate the role of acetylation process in erasing the expression pattern of lineage-specific genes and thus result in epigenetic reprogramming [14], [15]. All these changes reveal the importance of epigenetic control over stem cells differentiation. Therefore, histone acetylation modifications may afford us a novel strategy for tackling epigenetic puzzles, overcoming stem cell differentiation that interferes with final stem cell specialization and improving the efficiency of induced pluripotent stem (iPS) cell generation.

A simple and convenient way to manipulate epigenetic status is to use small molecules to interfere with epigenetic modifiers, such as histone acetyltransferase (HAT) [16], which can be targeted to specific regions of the genome and show varying degrees of substrate specificity, providing a dynamic, acetylation-based epigenetic code [17], [18]. It demonstrates an interesting phenomenon that the HAT proteins could be classified into several different subfamilies with little or even none sequence homology while sharing similar catalysis domain, which make them distinguished from other enzymes and thus worthwhile intensive study [5]. In addition, different HAT subfamilies have distinct catalytic specificities. To date, several typical transcriptional cofactors with HAT activity have been discovered, including GCN5, P/CAF, ESA1, CBP/p300 and Rtt109. Among them, GCN5, which belongs to GCN5-related N-acetyltransferase (GNAT) superfamily, was first found as a protein for amino acid biosynthesis in yeast [19], and then confirmed as a requirement for histone acetylation modifications. Previous bio-studies revealed that GCN5/PCAF family members showed great specificity for lysine 14 on H3 and relatively lower preference towards lysine 8 and 16 on H4 [20]. GCN5/PCAF family members could also acetylate non-histone protein, such as tumor suppressor p53 [21], the c-MYC oncoprotein [22], and the metabolic coactivator PGC-1α [23]. Moreover, protein complexes containing GCN5 also display a preference to H3 and H2B [24]. Owning to these interesting characteristics of GCN5, our theoretical studies on the acetylation mechanisms of GCN5 will be of great value.

To date, structures and functions of tGCN5 [25], [26] and yGCN5 [27], [28] have been extensively studied, while less theoretical studies have been performed on the structure and functions of hGCN5 which may possess more importance on disease researches. Recently, studies point out that GCN5 is tightly linked to aging and cancer due to loss of genome integrity [29] and closed to glucose metabolism disorder. Meanwhile, misfunction of GCN5 can lead to diabetes and aging problem [23]. GCN5’s involvement in superoxide-generating system also suggests its important role of regulation in immune response [30]. In human cells, CDC6 takes part in the formation of a complex with GCN5 containing Cyclin A-CDK2 and the following acetylation of CDC6 during S phase is reported to be essential for proper cell-cycle progression [31]. Moreover, studies on GCN5-containing complexes STAGA (SPT3-TAFII31-GCN5-L acetylase) [32], and TFTC [33] also indicate the importance of GCN5 in cancer research.

The deprotonation state of *ε*-amino group of the lysine substrate before reaction should be considered thus to give the high *pKa* values of the lysine residue. Generally speaking, glutamic and aspartic residue are two general bases which facilitate the deprotonation process [34], [35], [36], [37]. Several structural and mutagenesis studies related with GCN5 family members indicate that a conserved glutamic residue may be responsible for this process [27], [35], [36], [38]. However, crystal structure of hGCN5 [39] reveals that the location of this glutamic residue is beyond the proton transfer distance, while one strictly conserved water molecule among different GCN5 structures could form a continuous proton transfer pathway by hydrogen bonds (hbonds) called as “proton wire”, which had been observed in various biological systems [40], [41], [42], [43], [44], [45]. Besides, no other glutamic or aspartic residues have been found within 5Å distance of the reaction center.

The acetyl-transfer process is a key step in gene expression regulation. Consequently, the catalytic mechanism turns out to be the most fundamental question involved in all acetylation studies. Two distinct pathways in which acetyl-group transferred to lysine have been developed. One is ping pong mechanism, in which the acetyl-group is transiently attached to the enzyme and then to the amine substrate in the subsequent step [46]. The alternative mechanism, which is called as sequential mechanism, involves direct acetyl-group transfer from AcCoA to the amine acceptor. This process requires a formation of intermediate ternary complex of the enzyme, AcCoA and the substrate before catalysis [47].

**Figure 1:**
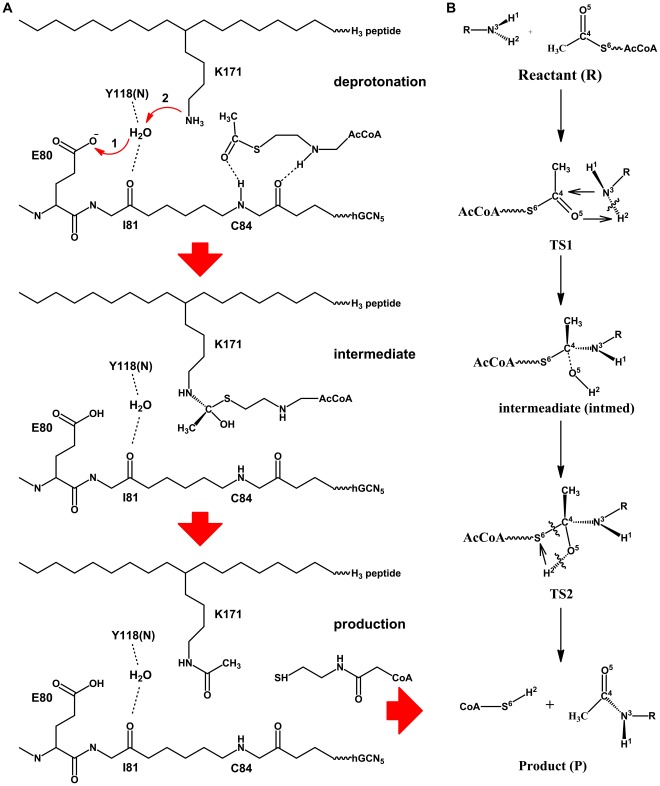
Proposed catalytic mechanism for GCN5/H3/AcCoA complex, (a) Three processes involved in the whole reaction, deprotonation, intermediate and production; (b) detailed mechanism for transition state and intermediate formation, critical atoms are numeric labeled for clarity.

Although most biochemical studies have assumed that GCN5 mediates the acetylation reaction using the sequential mechanism pathway [28], [36], [48], no crystal structures of the hGCN5/AcCoA/pH3 complex has been solved and a dynamic view of the catalytic process of hGCN5/AcCoA/pH3 and other HAT enzymes is still lacking. In this study, three steps were focused: 1) modeling a rational GCN5/H3/AcCoA complex structure based on known templates and mutagenesis experiments; 2) selecting reasonable structures based on the analysis of molecular dynamics (MD) simulations; 3) employing quantum mechanics/molecular mechanics (QM/MM) simulations to explore the detailed mechanism of acetylation process. The MD simulation results directly elucidated the specific role of residue Glu80, and WAT189 in proton transfer, Ile81 and Tyr118 in hbond interaction with WAT189 and Cys84 in polarization of carbonyl oxygen of AcCoA (CH_3_C(O)-), which are well consistent with findings in studies of GCN5 crystal structures [25], [28], [39], [49]. And snapshots for transition states clearly offer us a more convenient method to design proper regulators to control the global and local histone modifications. As illustrated in Figure 1A, three parts of the reaction pathway, deprotonation, intermediate ternary formation and production, were mainly studied. Sequential ordered (Figure 1B) proceeding through a ternary complex was closely investigated in our QM/MM simulations. Above all, our computational study may provide us a better insight into the acetylation reaction mechanism of HAT family, as well as suggestions for further experimental design and studies of substrate binding specificity.

## Results and Discussions

Prior to investigate the mechanism of catalytic process of GCN5 through computational method, it is of vital importance to obtain an accurate model of the enzyme-substrate complex. The initial hGCN5/AcCoA/pH3 complex structure was modeled on the basis of two homologous structures with high resolution, tGCN5 and hGCN5 (PDB ID code: 1PU9 and 1Z4R, respectively) [25], [39]. Then the model was refined based on the information from existing crystal structures and experimental data. Dynamic conformational changes were studied through MD simulations. Results were carefully compared with biochemical data for validation. Subsequent QM/MM simulations were performed on the complex system to investigate the mechanism of acetyl transfer from AcCoA to histone H3 peptide and to scan the potential energy for interactions between small molecule and enzyme in the acetylation process.

### MD Simulation Analysis for GCN5/AcCoA/pH3 Model

**Figure 2:**
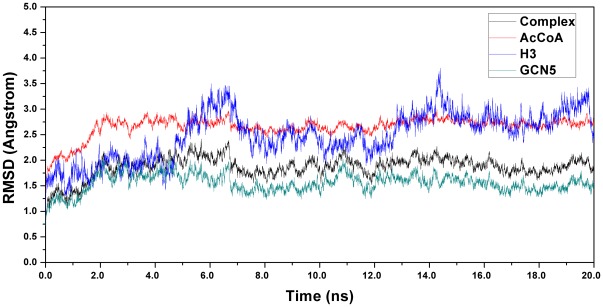
Time dependencies of the weighted root-mean-square deviations (wRMSDs) for the backbone atoms of GCN5/H3/AcCoA complex, AcCoA, H3 and GCN5 from their initial positions during the 20 ns simulation.

**Figure 3:**
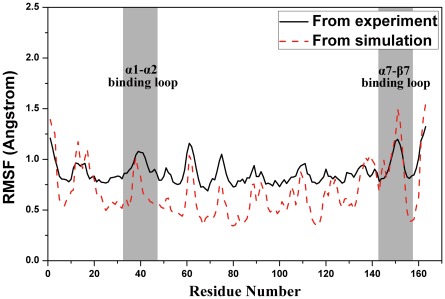
Residue fluctuations obtained by average residual fluctuations over the 20 ns simulation are illustrated in dashed lines, while the solid lines stands for the experimental results calculated from B factors of hGCN5 (PDB code: 1Z4R) crystal structure.

In order to remove a few inappropriate contacts in the structure from homologous modeling, MD simulation was performed to relax the system. In this section, a 20 ns MD simulation was carried out on the system. Root-mean-square deviation (RMSD) was monitored during the whole MD process (from *t* = 0ps), using the backbone coordinates of the model structure as the reference. As shown in Figure 2, the RMSD tends to be flat after 7 ns’ simulation, which indicates that structures located in this area theoretically be more reasonable. What deserves to be mentioned is that the RMSD of GCN5 part is only about 1.6Å and the reason leading RMSD of the complex to above 2Å is more likely to be the flexibility of H3 peptide and conformational change of AcCoA. Meanwhile, as the protocol of representing the stability of residues, the root-mean-square fluctuation (RMSF) of GCN5/AcCoA/H3 and GCN5/AcCoA was also calculated, respectively. B-factor of GCN5/AcCoA, as a measurement for any type of displacement of an atom from its mean position, was extracted from PROTEIN DATA BANK (PDB) website and was transformed to RMSF using formula 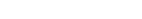. The consistency between theoretical and experimental RMSF in Figure 3 validates the rationality of our model. In details, the peak of RMSF curve apparently suggests the different loop regions of the protein. Loop α7-β7 (residues 144–154) of protein GCN5 is a highly conserved region among GCN5 families and has been reported as binding region [25], [26]. It was obvious that this region also differentiate most in our model, which indicated that this loop might change its direction to shape the binding pocket for substrate H3. Similarly, the location and displacement of loop α1-α2 also indicated its role in binding affinity with substrate H3. The other peaks appeared in Figure 3 correspond to the rest loops of the protein.

**Figure 4:**
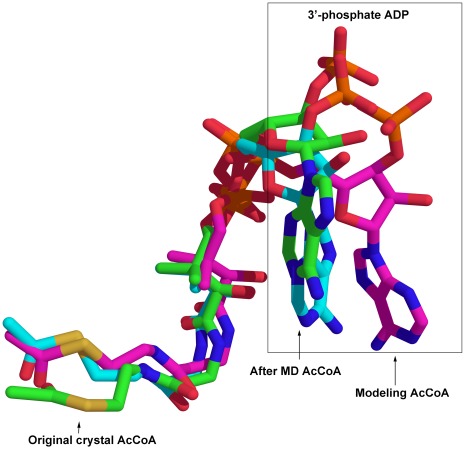
Conformation changes of AcCoA among original crystal structure, modeling structure and structure after MD simulation. The rectangle region corresponds to the 3′-phosphate ADP part of AcCoA, which implies the most different part among the three structures, indicating to transform into the appropriate conformation to provide the proper interactions with substrate H3.

In addition, the conformational change of AcCoA, which may provide appropriate reaction environment with hbonds and hydrophobic interactions, is also very important for catalytic reaction. The 3′-phosphate ADP conformation of AcCoA in hGCN5 (PDB ID code: 1Z4R) was substituted using the part in tGCN5 (PDB ID code: 1PU9) to offer a better hbond and hydrophobic function for substrate binding (see materials and methods). The RMSD of AcCoA using first modeling coordinates during 20 ns MD simulation is plotted in Figure 2, which evidently shows that the conformation of AcCoA fluctuates initially but becomes stable after 7 ns. Further analysis by superimposition of AcCoA with crystal structure (PDB ID code: 1Z4R), initial model and model after MD simulation also indicates that the most fluctuant part is the tail of 3′-phosphate ADP part, which is shown in Figure 4. From this result, it is clear that the final conformation is more reasonable due to some internal rearrangement, while further analysis via hbond and hydrophobic interaction would be discussed later.

It was reported that Glu122 of tGCN5 acted as the residue to accept the proton from lysine residue [49]. In our structure, a structurally conserved Glu80 is proposed for the similar role. Specifically, the Glu80, located at the cleft of the binding groove, is surrounded by several residues with hydrophobic side chains, including Phe70, Met72, Phe73, Phe78, Ile81, Val82, Phe115, Leu116, Tyr118 and Ile147, which increase the *pKa* value of Glu80 side chain and thus enable it as the acceptor of the proton [36], [49].

### Hbond Interactions Analysis

**Figure 5:**
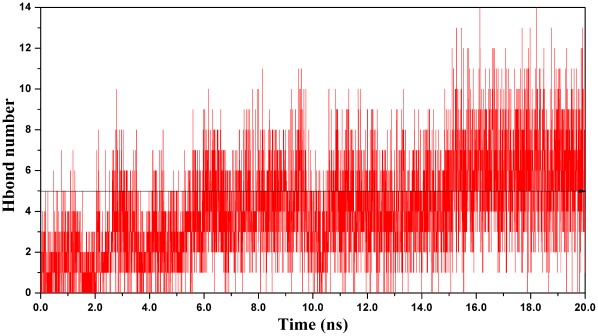
Number of average hbonds during 20 ns simulation.

**Figure 6:**
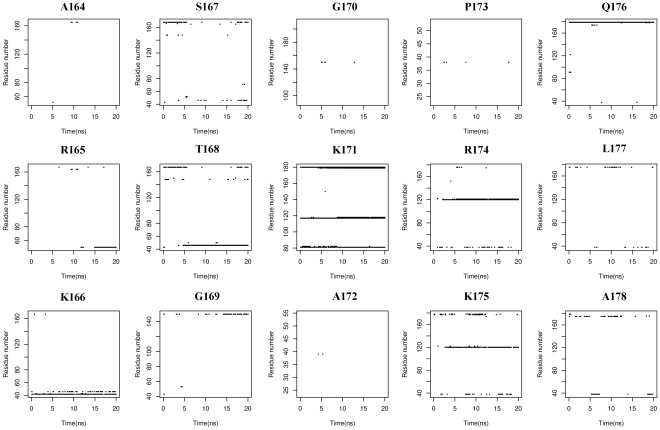
Time dependencies of hbond analysis for the whole H3 peptide showing critical residues for H3 tail binding and strong hbonds network for acetylation reaction center.

Because residues located in the hbond network are commonly validated for their critical roles in protein stability and catalytic process by bio-experiments, therefore, hbonds among substrate H3, AcCoA and GCN5 can offer us reliable analysis for interactions influencing binding affinity. As shown in Figure 5, an average of five pairs of hbonds exists between H3, GCN5 protein and AcCoA during the whole simulation. Detailed analysis of these hbonds is plotted in Figure 6, in which Lys171 of H3 forms six hbonds with the protein (Ile81, Val82, Thr117 and Tyr118), AcCoA and WAT180 (Figure S1, solid arrows, in Supporting Information), making it sufficiently stable for proton extraction and nucleophilic attack. Moreover, Lys166 and Gln176 of H3 display their particular importance for anchoring the histone peptide to the GCN5 protein, just as their counterparts, Arg8 and Gln19 in tGCN5 [25]. Integrating Figure 6 and Figure S1, Lys166 forms two hbonds with the Glu42 and Arg46 in α2 helix, while Ser167 and Thr168 are within hbond distance with Arg46 in α2 helix, respectively, which greatly implicate the participation of α2 helix in substrate H3 binding. In addition, a strong hbond connection between Gln176 and AcCoA suggests that AcCoA is also very important in H3 peptide tail binding. Moreover, Gly169, Arg174 and Lys175 also significantly interact with protein via tight hbonds with Tyr150, Arg38/Asp120/Glu121 and Arg38/Asp120, respectively. Therefore, these hbond interactions made the binding affinity of H3 stable enough for acetylation reaction process. WAT189, which is located in the middle of the amine nitrogen of Lys171 and the carbonyl oxygen of Glu80, forms two tight hbonds with Ile81 and Tyr118 of the protein. Meanwhile, this water molecule makes two additional hbonds with Lys171 and Glu80, providing a stable pathway for proton transfer. Overall, the stability of Lys171, WAT189 and Glu80 may facilitate proton extraction and transfer.

**Figure 7:**
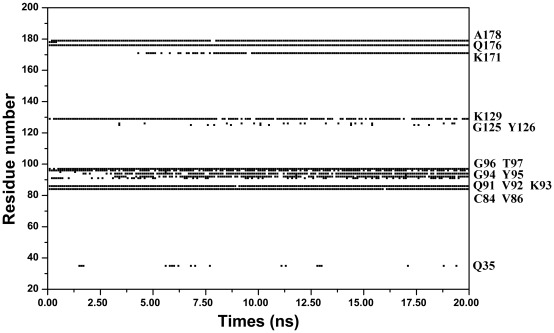
Residues involved in hbonds with AcCoA along the 20 ns simulation. Labels are listed for clarity.

The acetyl group donator AcCoA, in particular the pantetheine part, is also very important for catalytic process. As described in hGCN5 structure [39], the carbonyl oxygen of AcCoA thioester is hydrogen bonded to the backbone amide nitrogen of Cys84 of the protein. This hbond makes the carbonyl oxygen more electrophilic and thus lead to instability of C-S bond of AcCoA, which facilitates the bond rupture of C-S and bond formation of C-N. Other hbond interactions, as shown in Figure 7, also contribute the tight binding of AcCoA with GCN5 protein and substrate H3.

### Hydrophobic Interactions Analysis

**Figure 8:**
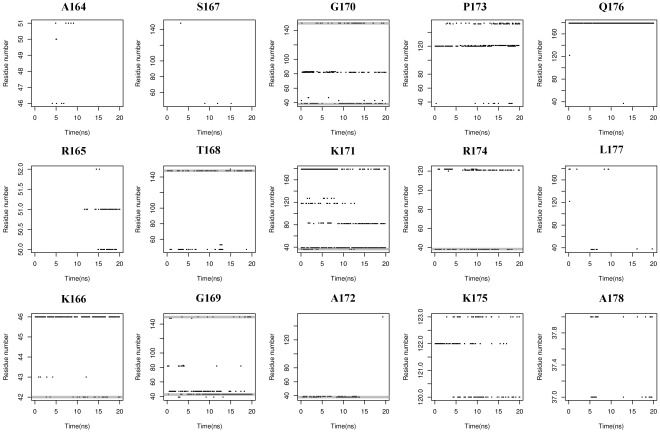
Time dependencies of hydrophobic analysis for the whole H3 peptide, suggesting the recognition of G-K-X-P sequence (Gly170-Pro173) and the importance of loop α1-α2 (Arg38 and Met39, in gray regions of Gly170, Lys171, Ala172 and Arg174), α2 helix (Glu42 and Tyr43, in gray regions of Lys166 and Gly169) and loop α1-β7 (Lys148 and Tyr150, in gray regions of Thr168, Gly169 and Gly170) for substrate binding.

**Figure 9:**
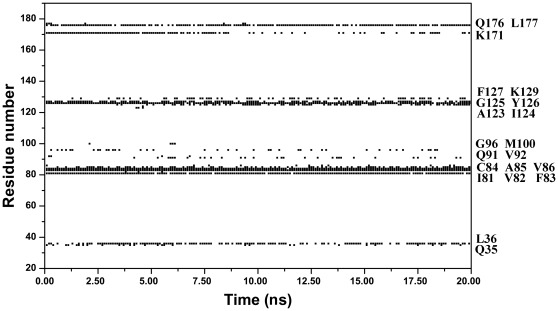
Residues in hydrophobic interactions with AcCoA along the 20 ns simulation time series. Labels are listed for clarity.

As another important function, hydrophobic interaction provides a favored nucleophilic attack environment. It has been reported that, in tGCN5, most hydrophobic contacts are located in the middle part of the histone H3 peptide, which are called as G-K-X-P recognition residues [25]. Our structure is also consistent with this feature, showing extensive hydrophobic interactions along residue 170–173. Details of these interactions are plotted in Figure 8 and Figure S1 (dashed arrows, in Supporting Information), showing how these interactions are conserved during 20 ns MD simulation. As shown in Figure 8 and Figure S1, residues involved in these interactions are located at loop α1-α2 (Arg38, Met39), α2 helix (Glu42, Tyr43), β4 sheet (Val82) and loop α1-β7 (Lys148, Tyr150), demonstrating the importance of loop α1-α2, α2 helix and loop α1-β7, which is well consistent with hbonds analysis and works before. According to Figure 9, AcCoA forms several hydrophobic interactions with GCN5 and H3, which helps to locate it into a better reaction environment. Meanwhile, an interaction between Gln176 and AcCoA is also displayed in Figure 9. As shown, the tail of the 3′-phosphate ADP part of AcCoA sits beside Gln176, completely being parallel with the side chain of Gln176 (-CH_2_-CH_2_-C(O)-NH_2_), which provides strong hydrophobic effect for H3 tail binding. With the aforementioned hbond interaction between Gln176 and AcCoA, it is believed that the internal rearrangement of 3′-phosphate ADP part discussed in MD part is indeed a necessary for the binding affinity of H3 peptide.

### QM/MM Results

Since the reliability of our model has been proved by hbond and hydrophobic interaction analysis, it is possible to further explore the mechanism of acetylation in detail. Analysis of conformations from MD trajectories does not find any evidence for the ping-pong mechanism, which is in accordance with previous experimental studies [28], [36], [48]. Thus, the sequential mechanism is mainly discussed. According to Figure 2, the RMSD is most flat between 12 ns and 18 ns. Two criteria were considered for QM/MM initial structure selection: structure located around the middle point of the region (15–16 ns), and proper distances for atoms involved in proton transfer and nucleophilic attack process (distance between C4 and N3 was less than 3.6 Å). According to our simulation, the fluctuation of distance between C4 and N3 tended to be flat after 10 ns, and the average distance was about 3.5 Å. Finally, four structural snapshots at 14492ps, 15212ps, 15840ps and 16156ps were extracted from the MD trajectory and selected for QM/MM calculation. Although the calculation for the structure at 14492ps failed for final convergence to give a conclusive result, the other three structures showed similar results with only differences in energy barriers, theoretically suggesting the same catalyzing mechanism in our study. Since the relative energy barrier for the structure at 15212ps was more favored, further discussion of the QM/MM results is mainly focused on this structure.

All QM/MM simulations were performed using ONIOM method encoded in Gaussian03 described in materials and methods. As for QM layer atoms selection, those proved to be critical for proton transfer and acetyl transfer are considered, including atoms involved in hbond interaction with Lys171, WAT189 and AcCoA. Molecular mechanical Amber force field is used to characterize the rest atoms.

In principle, residue lysine is positively charged in physiological environment thus must be undeprotonated before nucleophilic attack. Structural analysis suggested the key residue Glu80 for the acceptance of this proton. Structure obtained from MD simulations was optimized using ONIOM method firstly. The O-H (WAT189) distance is elongated from 0.96 to 1.13 Å and N-H (Lys171) changes from 1.01 to 1.06 Å, which results in the bonds break of O-H/N-H and thus bring the system into a transition state (TS) of deprotonation process. After another optimization using TS structure, the proton of Lys171 is successfully transferred to Glu80 mediated by WAT180 to form the product, which should be the initial reactant of acetylation reaction. As calculated, a potential energy barrier of 0.229 kcal/mol is required for product/reactant formation, which is relatively low for proton transfer reaction process and thus the substance could easily get accessible to the unprotonated state.

The most concerned problem is the mechanism, under which the substrate react with AcCoA. Crystal structures of GCN5 and its related homologues also intuitively indicate the need of the formation of a ternary complex of GCN5, AcCoA and substrate [49], [50], [51]. Biochemical data accumulated from kinetic and mutational experiments [26], [27], [36], [52], however, only addressed the mechanistic details based on structures and activity analysis. However, none theoretical studies have been reported to describe the intermediates and the associated energy barrier to date. Therefore, our endeavors turn to the hotspot of transition state point and the required reaction energy barrier, which may be applied for further investments of regulators against HAT enzymes.

All the atoms involved in the acetylation process are defined and labeled in Figure 1B. Classical chemical theory of acetylation reaction favors a mechanism, in which the hydrogen (H2) of Lys171 first transfers to the oxygen atom (O5) of CH_3_CO- part of AcCoA, forming an intermediate (Figure 1B, TS1 and intermediate). Then the hydrogen (H2) quickly migrates to atom S6, which results in the ruptures of the bonds of O5–H2, the recovery of carbonyl bond between C4 and O5, and the release of CoA. Before and after the formation of intermediate state in the whole reaction process, two similar four-member ring structures nearly planar are formed in TS1 and TS2, while atom C4 experiences hybridization changes of *sp^3^-sp^2^-sp^3^*. In those four member ring structures, two small angles less than 80° are detected (C4O5H2-72.5° and C4N3H2-78.4° for TS1, C4S6H2-53.7° and S6C4O5-79.5° for TS2), which bring large strains to the TS system and make it unstable.

The energy profile was chosen as the criterion to determine the reaction pathway, along which, the energies of reactant (R), transition state (TS) and product (P) were scanned though two-dimensional QM/MM potential energy surface, by defining the distance between atoms as the reaction coordinates.

**Figure 10:**
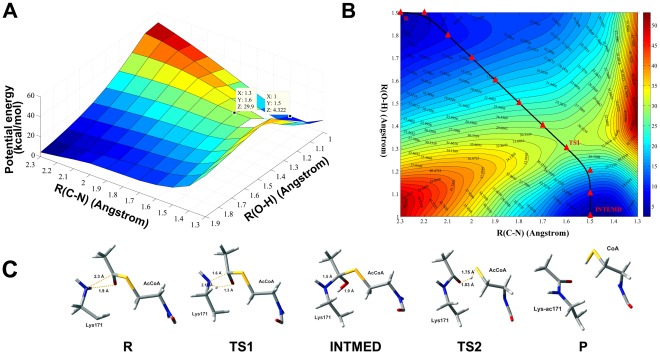
The potential energy surface (PES) of reaction pathway for GCN5/H3/AcCoA complex. (A) Energy barriers of transition state (TS1) of addition process and intermediate production (INTMED) are marked along the defined reaction coordinates. (B) Contour plot of the PES corresponding to (A). The pink triangle line represents the lowest energy path according to the calculation of PES, positions of reactant (R), transition state 1 (TS1) and intermediate product (INTMED) are also displayed. (C) Critical structures along the reaction coordinate. Information of bonds formation and rupture is displayed and labeled in yellow dashed lines.

As for sequential mechanism, R(C4–N3) and R(O5–H2) were used as the reaction coordinates. Energy profile variation was monitored as the defined distances were progressively decreased. Theoretically, transition state should be the stagnation point of the energy surface while production state should go to the local minimum point. According to the calculation, the overall relative potential energy barrier is about 29.9 kcal/mol for TS1 and 4.322 kcal/mol for intermediate product (INTMED). The structure of the transition state (TS) pathway is determined by adiabatic mapping at the QM/MM level (Figure 10A, B). Along the reaction pathway, the TS1 state is located as R(C4–N3) = 1.6Å and R(O5–H2) = 1.3Å, which falls into their covalent bond distances; while the distance R(N3–H2) = 1.28Å, which is much longer than its covalent bond distance and thus lead to rupture of this bond. After the intermediate is formed, the distance R(S6–H2) quickly decreases into 1.75Å in TS2, while the distance of R(O5–H2) increases from 1.0Å of INTMED to 1.03Å. As R(S6–H2) continually decreases, the R(O5–H2) becomes much larger (1.91Å), which finally causes the rupture of O5–H2 bond to form the final products (P). Bonds formation and rupture during the critical structures for different reaction states are illustrated in Figure 10C to clearly demonstrate the catalytic process of the system.

**Figure 11:**
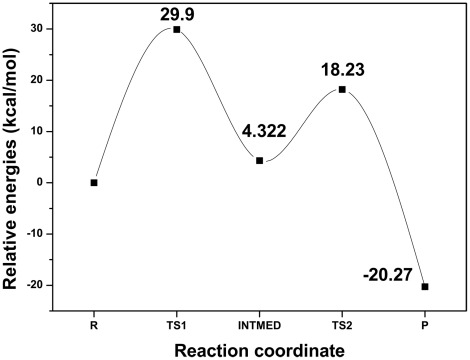
Whole relative energy profile along the reaction coordinate, reactant (R), transition state 1 (TS1), intermediate product (INTMED), transition state 2 (TS2) and final product (P). Relative energy barriers are labeled for each state.

Similarly, the energy shift of INTMED-P process was also monitored as the highest point of the reaction path. The energy profile is plotted in Figure 11 along the whole reaction coordinates. The relative energy barrier of this process is 18.23 kcal/mol while the product relative energy equals to −20.27 kcal/mol, which is much less than the reaction state, suggesting the stability of forming product. Moreover, results of QM/MM simulation, especially specificities between small molecule and enzyme in TS structures, clearly offer us a convenient insight on the discovery of regulators against GCN5 and its related HAT family proteins.

### Conclusions

As aforementioned, GCN5 has been reported to play critical roles in metabolism, DNA repair, oxidation system, transcriptional silencing, genome stability and so on. However, rare theoretical simulation and mechanism studies have been reported, which greatly hamper the fundamental insights of the dynamic acetylation. Therefore, in the present study, homologous modeling method was employed to construct the GCN5/AcCoA/pH3 system by using tGCN5/hGCN5 as the templates. A 20 ns MD simulation was carried out to obtain a stable and proper structure. Existing hbond network, hydrophobic interactions and key residues involved in catalytic reactions based on experimental data and homology crystal structures were used as the validation sources. Structural analysis reveals that Glu80, WAT180 and Lys171 form a “proton-wire” for the deprotonation process of Lys171. Moreover, RMSD and RMSF analysis suggest that both loop α7-β7 and loop α1-α2 play a critical role in binding substrate H3. While hbond and hydrophobic interactions analysis afford a deeper understanding on the key residues to stabilize the proton wire transfer, the proton receptor Glu80, the reaction active site Lys171 and the residues involved in substrate binding, such as particular importance of Lys166 and Gln176 for anchoring the histone peptide to the GCN5 protein. QM/MM simulation was carried out for further acetylation mechanism investigation, from which energy barriers for the entire acetylation reaction were obtained, and structures from different reaction states were analyzed in detail, suggesting proposed mechanism of the formation of ternary complex.

**Figure 12:**
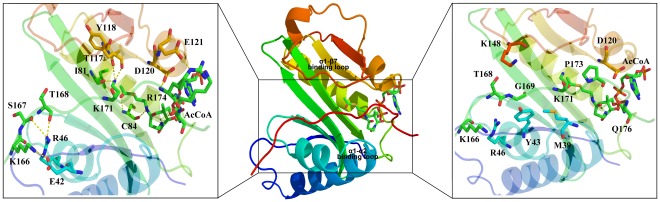
Overall structure of GCN5/H3/AcCoA complex and the interactions. Two important loops involved in H3 binding, α1-α2 loop and α1-β7 loop are illustrated. Hbond interactions indicate those residues in hydrogen bonds with H3, which are displayed in yellow dashed lines in left enlarged figure; hydrophobic interactions presented in right enlarged figure indicate those residues forming hydrophobic interactions with H3 to provide appropriate reaction environment.

**Figure 13:**
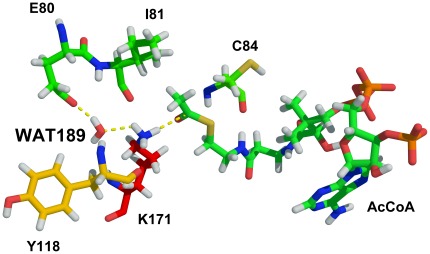
Critical residues (atoms) for QM region in QM/MM calculation. Hbonds involved in deprotonation and acetylation reaction paths are labeled in yellow dashed lines.

In summary, our research provides a detailed histone acetylation mechanism for GCN5/AcCoA/pH3 system, and explores the transition states for the entire reaction process, rendering useful implications for the discovery of regulators against GCN5 enzymes. Furthermore, results from the present study will offer theoretical basis for the mechanism studies on other HAT family proteins.

## Materials and Methods

### Molecular Modeling

To date, several crystal structures (PDB ID code: 1QSN, 1PU9, 1Z4R [28], [49], [51]) have been solved, which either contain H3/H4 or AcCoA or is a ternary complex containing H3/H4 and CoA, but none of these structures contain both H3 and AcCoA, which should be originally used for fully dynamic investigation of the acetylating mechanism. Starting model of the GCN5/AcCoA/pH3 complex system was constructed on the basis of structure of hGCN5 [39] (PDB ID code: 1Z4R) solved at 1.74Å, which contains GCN5 and AcCoA. Crystal structure of hGCN5 shows that the HAT domain is composed of a mixed α/β topology including seven α-helices and seven β strands. Two loops, one locating between α1 and α2, the other preceding β7, are critical for interactions of binding affinity [25], [26], [35]. These two loops, which are located on the same surface of the domain, shape a cleft directly situated above the acetyl-group of AcCoA and for potential the catalytic region of histone H3, proving a suitable environment for acetylation. To our knowledge, the complex structure after H3 binding should show some pronounced rearrangement of C-terminal segment and wider substrate binding grooves, which should be remodeled beforehand. Moreover, based on the analysis of two tGCN5 structures (PDB ID code: 1QSR and 1QSN) [49], it is considered that the ligand AcCoA may has a conformational change in 3′-phosphate ADP part to provide specific substrate tail binding of H3. Thus, this section is a control at how to merge the substrate H3 into the GCN5/AcCoA system and make some pretreatments for the next step. Overall, the following points should be paid more attention in model construction: 1) the H3 peptide lies in the binding pocket with interaction of GCN5 and AcCoA, H3K166 and H3Q176 are two residues that play particularly important role for anchoring the histone peptide binding with GCN5, 2) The N atom of residue lysine is designed to lie within 3.5 Å to the water molecular and 3.5 Å to C atom of AcCoA, 3) AcCoA conformation change to provide hydrophobic environment for H3 tail binding, 4) loop α7-β7 rebuilding to open the pocket for H3 binding. So first the α7-β7 loop was replaced with the one from structure of tGCN5 (PDB ID code: 1PU9) using Homology module of InsightII (Accelrys, San Diego, CA), peptide H3 was extracted from structure of tGCN5, then merged into reconstructed modeling structure, and the 3′-phosphate ADP of AcCoA was replaced with the one from tGCN5. At last, the reconstructed AcCoA was merged into the modeling structure again to form the full GCN5/AcCoA/pH3 system. The initial structure was minimized by using Sybyl software package (Tripos, St. Louis, MO) with following setups: 1) a distance-dependent dielectric function, nonbonded cutoff of 8Å, 2) amber charges were assigned to protein while Gastieger-Hückle charges are given to AcCoA, 3) system minimization until none atom collisions were found. Final structure of the complex is shown in Figure 12.

### Molecular Dynamics Simulation

Before MD simulations, charge information of molecular AcCoA was calculated by using RESP method encoded in the AMBER suite programs (version 10) and then was put into Gaussian software for calculation at HF/6-31G*. Then the complex system was reloaded into AMBER after the application of AMBER Parm99 force field, and 9 counter ions were added using 1Å grid to neutralize the system, finally suitable sized box with 8Å water TIP3PBOX was loaded into the system to form the protein environment. In together, the prepared system had 17950 atoms, including 4979 water molecules. After these preparations, energy minimization was carried out to remove inappropriate contact. MD simulations were performed with nonbonded cutoff of 8Å, integration step of 2fs time interval and SHAKE algorithm [53] was employed to constrain all covalent bonds including hbonds. The system was first to heat up to 300 K for 50 ps with the protein complex fixed, and then in equilibrium with constant temperature and pressure (NPT) under periodic boundary conditions. Particle mesh Ewald method was applied to calculate the long-range electrostatics interactions. The program LIGPLOT version 4.4.249 was used to calculate the hbond and hydrophobic interaction between the protein and the substrate, and an appropriate structure was extracted in accord with experimental data to offer an optimized one for QM/MM calculation.

### QM/MM Simulation

All simulations were carried out with versions of Gaussian03. The hybrid QM/MM mechanical approaches [54] have been widely employed to enzymatic reaction mechanisms studies [55], [56], [57], [58], [59], [60]. Among them, two-layered ONIOM method encoded in Gaussian03 has long been proved its availability in large system and manageability of computation costs [61], [62], [63]. In two-layered ONIOM method, the whole molecule is divided into two parts, the inner subsystem including enough and essential atoms for reaction and the outer part comprises the rest. The total ONIOM extrapolated energy is defined as [64]:
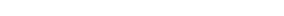



The subscript “model” refers to the inner subsystem and link atoms, while “real” is defined as the full system. Impact of low level method on outer part is obtained by D-value between “real” and “model”. Together with the energy of “model” obtained via high level method, the final ONIOM energy is formed. Totally, computational complexity is reduced on the premise of accuracy by perform high level calculation just on critical region.

The QM system contained 71 atoms, including the part of Glu80, Ile81, Tyr118, Lys171 and WAT189, and CH3-C(O)-S-CH2-CH2-NH-C(O) part of AcCoA, which is shown in Figure 13. Other atoms of the complex were classified into MM system. Besides, QM boundaries were treated with the link atom approach [65], by introducing H atom to saturate the valence of QM boundary atoms. And QM was treated by using hybrid density functional theory (DFT) of B3LYP (6-31G(d)) level for its favorable computational effort/accuracy ratio, while AMBER Parm99 force field was used for MM part. The reaction energy profile was evaluated by single point energy calculations at B3LYP QM/MM level.

## Supporting Information

Figure S1 **Summary of substrate H3 interactions.** The solid and dashed arrows represent the residues of the GCN5 and AcCoA within hbond and hydrophobic distance of the peptide residues, respectively.
(TIF)
