# Supplementary figures and images for: Investigation of the Acetylation Mechanism by GCN5 Histone Acetyltransferase
Source: PLoS One. 2012 May 4;7(5):e36660. doi: 10.1371/journal.pone.0036660 (PMC3344931; doi:10.1371/journal.pone.0036660)

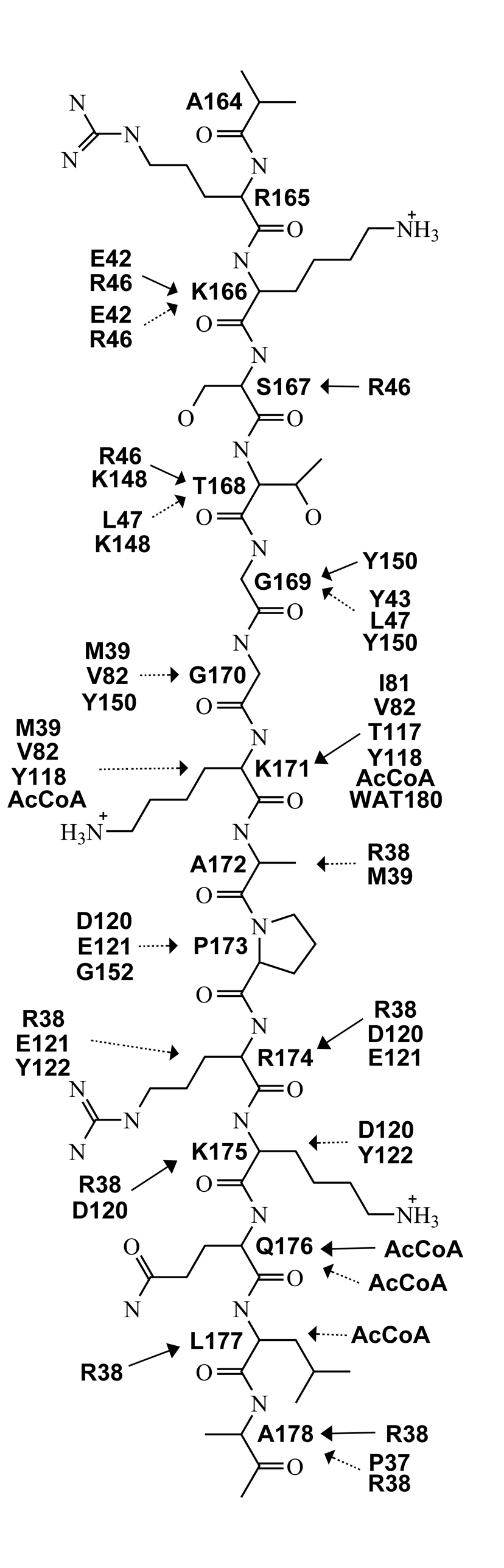

Supplement: Figure S1 — Summary of substrate H3 interactions. The solid and dashed arrows represent the residues of the GCN5 and AcCoA within hbond and hydrophobic distance of the peptide residues, respectively. (TIF) [file pone.0036660.s001.tif]
